# Inclusivity in education for autism spectrum disorders: Experiences of support from the perspective of parent/carers, school teaching staff and young people on the autism spectrum

**DOI:** 10.1080/20473869.2022.2070418

**Published:** 2022-05-09

**Authors:** Laurence Hasson, Saskia Keville, Jen Gallagher, Dami Onagbesan, Amanda K. Ludlow

**Affiliations:** 1 Department of Psychology, Sport and Geography, University of Hertfordshire, Hatfield, Herts, United Kingdom; 2 Islington Child Adolescent Mental Health Service, Wittington Health NHS Trust, London, United Kingdom

**Keywords:** Autism spectrum disorder, inclusivity, education, support, children and young people, teachers, parent/carers

## Abstract

Inclusive practices mean many children with autism spectrum disorders (ASD) attend mainstream education settings. To manage the stressors involved and access its benefits, support can be critical. Indeed, insufficient support can detrimentally impact wellbeing, longer-term development, and the inclusivity agenda. Expanding a limited evidence-base on educational support after diagnosis, focus groups and interviews were conducted for eight parent/carers of children with ASD, twelve special education needs (SEN) school staff, and four children with ASD attending mainstream school. An inductive thematic analysis on the data elicited three themes: a system overwhelmed by unmet needs, the impact on quality of life, and hope for the future. The overwhelming finding was a significant lack of education support for parent/carers and school staff, with the mainstream education system poorly designed and insufficiently resourced to facilitate the inclusion of children with ASD, particularly for those impacted by historic difficulties with access. The tireless work of parent/carers and frontline SEN educators fostered a sense of hope and engendered inclusivity for the children who participated, who felt supported. Given their buffering role, protecting and supporting parent/carer and SEN teacher wellbeing requires a policy shift supporting longer term inclusivity alongside improvements in funding streams and accessibility in provision.

Autism spectrum disorder (ASD) is a lifelong condition characterised by social communication difficulties and repetitive behaviours (American Psychiatric Association 2013). The standardised prevalence is around 1.76% of children within schools in England (Roman-Urrestarazu *et al.* 2021); and there is a high incidence of mental health difficulties (Murphy *et al.* 2016). Whilst obtaining a diagnosis of ASD can be a lengthy, difficult process (Crane *et al.* 2018), less is known about experiences of subsequent support after receiving a diagnosis. Issues with access are indicated with 70% of adults with ASD believing they receive inadequate provision from social services and would feel less isolated with more support (Bancroft *et al.* 2012). Further, compared to 80% of the general population, only 32% of autistic individuals are in paid work, impacting their lives (National Autistic Society (NAS), 2016). Support is important for families of people with ASD; parents of children with ASD generally have increased parenting stress, higher levels of anxiety and depression, higher unemployment and financial difficulties, and overall lower quality of life (Dillenburger *et al.* 2015; Vasilopoulou and Nisbet 2016).

Early support following a diagnosis of ASD is considered essential for improving quality of life and reducing parental stress (Keen and Rodger 2012), potentially mitigating some of the psychosocial and financial difficulties faced by autistic individuals and their families. Yet, support tends to be limited, difficult to access, insufficient and unsatisfactory (Crane *et al.* 2018; Galpin *et al.* 2018). Many people emphasise a fight to obtain support (Hebron and Bond 2017), with parents battling with services (Galpin *et al.* 2018; Preece 2014). Many professionals also wish they could offer greater support (Rogers *et al.* 2016; Unigwe *et al.* 2017) particularly as adapted educational provision in schools is difficult to access for children with ASD (Tissot 2011). This difficulty is exacerbated by a lack of understanding and awareness about ASD (Galpin *et al.* 2018; Preece 2014; Roberts and Simpson 2016), in part due to diverse and differing needs (Donnellan, Hill, and Leary 2012).

In United Kingdom (UK) schools, of the 120,000 pupils with ASD, 72% attend mainstream school (Department for Education 2018b), yet, according to parents, 74% of children do not attend a school which fully supports their child's needs (NAS 2021). Indeed, in English schools the exclusion rate of children with ASD is twice the rate of those without special education needs (Department for Education 2018a); clearly schools inadequately understand or accommodate the needs of children with ASD. Furthermore, children with ASD are more likely than other children to be victims of bullying (Humphrey and Hebron 2015), which can impact on wellbeing. Over 60% of mainstream teachers in England believe their initial training did not adequately prepare them to teach children with ASD (NASUWT 2013), with many believing they lack the training to appropriately meet their needs (Symes and Humphrey 2012; Anglim, Prendeville, and Kinsella 2018; Lindsay *et al.* 2013). Other research paints a consistent picture: just a quarter of children with ASD felt happy or included at school, one in four parents said it took more than three years to set up the right support for their child (NAS 2021) and 70% were dissatisfied with teachers’ level of understanding of ASD (NAS 2017). Furthermore, parents of children with ASD find it disproportionately difficult to obtain appropriate educational provision for their children compared to families of children with other disabilities (Parsons, Lewis, and Ellins 2009).

Whilst there is a drive for inclusivity within education for children with ASD through adapting provision (Humphrey and Symes 2013; Lindsay *et al.* 2014), funding the provision of support is a major challenge for schools often due to wider systemic structures. For example, 82% of mainstream schools do not have sufficient funding to adequately provide for pupils with special educational needs (SEN), and 89% of school leaders report cuts to local authority services negatively impacts provision (The Key 2016a). To ensure equality and inclusion within mainstream education it seems critical to address levels of support for children with ASD, which is likely to involve wider issues. Indeed, from an ecological systems standpoint there are multiple and differing layers which surround individuals in general (Bronfenbrenner 1992); for children with ASD this ranges from home contexts, to peers, to schools, to healthcare, to areas beyond their awareness involving local authorities, socio-political systems and wider societal discourses. Some local authorities have recognised the specific needs of children with ASD or other additional needs, funding specialist provision within mainstream settings; with its positive impact evaluated through a consideration of these systemic layers (Hebron and Bond 2017). Yet, these areas seem less understood within generic mainstream provision.

The aim of this study was to allow children with ASD, parents/carers of children with ASD and SEN school staff to voice their experiences of support in mainstream education. The project aimed to facilitate understanding of people’s experiences, and identify issues, challenges and examples of good practice.

## Materials and methods

### Research design

As an exploratory study a qualitative reflexive thematic analysis was used to explore the common themes of provision for ASD from the perspective of parent/carers, children themselves, and special educators (Braun and Clarke 2006; 2019). Key steps were conducted to ensure validity and credibility (Treharne and Riggs 2015; O'Brien *et al.* 2014) and inclusive research was implemented throughout the study (Walmsley, Strnadová, and Johnson 2018). Firstly, to ensure the research was accessible, understandable, and explored relevant areas, several autistic children/young people and parents of children with ASD were involved in the design of the research, and recruitment posters. These consultants did not partake in the research interviews.

### Participants

This research took place in an UK inner-city borough with a diverse and high-density population. To facilitate triangulation (Treharne and Riggs 2015) there were three study populations: children and young people with ASD, parents/carers of children with ASD, and school staff working with children with ASD. A purposive sample was recruited through snowballing; parent/carers were recruited through the local parent support group for SEN, and school staff through emails sent to the borough’s SEN Coordinators (SENCO). Children were recruited through school staff and parents who explained the study to each child and shared the participant information sheet. All responders met the inclusion criteria of having or teaching a child with ASD or having ASD and attending mainstream school. Demographic data (Table 1) was collected for:

**Table 1.** Participant demographics.

**Participants** **Group** **(*N* = 24)**	Participant code	Mainstream Education setting	Group format	Length of interview	Gender	Age	Ethnicity; first language	Number of children;	Details of children with ASD	Other information
**^5^Parents (*N* = 8)**	Parent 1	Primary	Individual	Approx. 60 mins	Female	42	White British; English	4	Son Age 8 Diagnosed age 5; co-occurring speech and language difficulties, seizures	
	Parent 2	Primary	Group 1	120 mins	Female	50	White British; English	3	Son age 11; Diagnosed age 10	
	Parent 3	Primary	Group 1	–	Female	44	White British; English	3	Daughter age 9; diagnosed age 7; co-occurring ADHD, sensory processing difficulties, Hypermobility)	Parent of CYP1
	Parent 4	Primary	Group 1	–	Female	26	White British; English	1	Son age 7; diagnosed age 2yrs 10 months; co-occurring Anxiety; hypermobility)	
	Parent 5	Primary	Group 1	–	Female	43	White British; English	3	Daughter age 7; diagnosed age 4	
	Parent 6	Secondary	Individual	Approx. 60 mins	Female	45	White British; English	2	2 sons with ASD: age 17 (diagnosed age 3; co-occurring global learning disability); age 14 (diagnosed age 9; co-occurring ADHD, dyspraxia)	Parent of CYP2
	³Parent 7	Secondary	Group 2 / individual	(Group2: 67 mins; individual: 64 mins)	Male	57	White British; English	_²	Granddaughter (NB: identifies as non-binary) age 15; Diagnosed age 8; co-occurring epilepsy)	
	Parent 8	Primary & secondary	Group 2 / individual	(Group2:as above; individual: 66 mins)	Female	40	Pakistani; Urdu	4	2 children with ASD age 14 (son diagnosed age 14; ADHD) and daughter age 5; diagnosed age 5; co-occurring anxiety)	
**^5^Staff (*N* = 12)**	SENCO 1	Secondary	Group 3	Group3: 93 mins	Male	36	White British	–	^4^11-16	Time in role: 5 years
	SENCO2	Primary	Group 3	–	Female	29	White other	–	^4^3-11	Time in role: 1 year
	SENCO 3	Secondary	Group 3	–	Female	63	White British	–	^4^11-16	Time in role: 17 years
	SENCO 4 – inclusion manager	Primary	Group 3	–	Female	46	English	–	^4^4-11	Time in role: 2 years
	TA1 –	Primary -higher level teaching assistant	Group 4	Group4: Approx. 60 mins	Female	33	White British	–	^4^11-16	Time in role: 8 years
	TA2	Primary – nursery nurse	Group 4	–	Female	29	White British	–	^4^4-5	Time in role: 2 years
	TA3	Secondary	Group 4	–	Female	21	Black British Caribbean	–	^4^5-7	Time in role: 2 weeks
	TA4	Primary	Group 5	Group5: Approx. 60 mins	Female	33	_²	–	^4^6-11	Time in role: 6 years
	Teacher1	Primary	Group 5	–	Female	33	British	–	^4^7-10	Time in role: 9 years
	Teacher2	Primary	Group 5	–	Female	34	White British	–	^4^3-11	Time in role: 2 years
	Teacher3	Primary	Group 5	–	Female	24	British	–	^4^5-10	Time in role: 2.5 years
	Assistant Head	Primary	Group 5	–	Female	_²	_²	–	_²	_²
**¹^&5^Children and Young People (*N* = 4)**	CYP1	Primary	Individual	Between 30-60 min	Female	9	White British; English	–	Diagnosed Age 7 (co-occurring sensory processing difficulties, ADHD)	Child of Parent3
	CYP2	Secondary	Individual	Between 30-60 min	Male	14	Identified White English/Scottish; English	–	Diagnosed Age 9 (co-occurring ADHD, dyspraxia)	Child of Parent6
	CYP3	Secondary	Individual	Between 30-60 min	Male	11	Bangladeshi; English	–	_²	
	CYP4	Secondary	Individual	Between 30-60 min	Male	15	British; English	–	Diagnosed Age 10	

^1^Diagnosed with ASD.

^2^A hyphen indicates that the participant did not answer these questions when requested.

³Grandparent who was primary carer.

^4^Age ranges of autistic children staff worked with.

^5^The term ‘parent’ will be used to denote parent or grandparent primary carer; ‘staff’ to denote school SEN teaching staff; and ‘child(ren)’ or ‘young person’ to denote the young people involved in the study or the children referred to by parents and staff participants.

- twelve school SEN teaching staff (assistant head (n = 1), SENCO (n = 4), class teacher (n = 3), teaching assistant (TA)(n = 4) (mean age: 34.6; age range: 21-63)
- seven parents and one grandparent who were primary carers of ten children with ASD (mean age: 43.4; age range: 26-57; mean age of child with ASD: 10.7; eight of the children had additional needs)
- 4 children with a diagnosis of ASD (mean age: 12.3; age range: 9-15; one with co-occurring attention deficit hyperactivity disorder (ADHD) and sensory processing difficulties)

### Instrument

The interview schedule was developed by the research team for each population group through issues raised in the literature. To ensure inclusive research with accessible and understandable interview questions, several people within each of the three population groups were consulted on the design of the interview schedule, with all suggestions implemented. This included four children with ASD and their parents. These consultants did not partake in the research interviews.

The interview schedule was used to guide the interviews, with each question asked verbatim to each population group; all questions were open allowing for positive and negative responses; prompts and follow up questions were used, if necessary, to allow a more nuanced exploration of individuals’ lived experiences. For all groups there was an initial building of rapport phase, which involved providing details about the interview; children were additionally asked a generic question about likes and dislikes about school to start the dialogue.

The children were asked if they had ever had any difficulties at school; what help they hoped to get; what support they received; what was good or not good about it; how the support made them feel if they had it, or did not have it; if there was anything that could have been done differently to support them at school and elsewhere; what they had learned from the support given.

Parents were asked about what was helpful and unhelpful about the support they received; if the support was as expected; challenges and help in accessing support; whether changes were needed or not; feelings about access, and any impacts from the process of obtaining support; advice they would offer to others.

School staff were asked about the typical support available through the school for young people with ASD; who provided that support; the support available to parents/carers of young people with ASD; the expectations of parents about the support offered to their child; the helpful or unhelpful kinds of support on offer; what support was easier or harder to obtain; the staff’s specific role in offering support; if they felt supported or if there were any restrictions in providing support; support they could use to benefit their practice; if there were potential barriers to providing support to young people with ASD; if there were any systemic changes needed to help improve provision; tips for best practice in the area; what they had learned from the experience of supporting young people with ASD.

All interviews concluded with how they felt talking about this and if they would like to add anything more.

### Data collection

The first author, a clinician experienced in working with diverse population groups, conducted all interviews face-to-face before the COVID-19 pandemic, either on school premises (school staff and children) or at the local support group premises (parent/carers). These premises were used to increase accessibility to the research for participants. Focus groups were conducted for staff and parent/carers; due to necessity and to ensure accessibility, ecological adaptations were made according to the number of people who came to the groups. For example, two parent/carers completed planned focus groups as individual interviews; two parent/carers started a focus group and completed the interview individually. All children at secondary school were interviewed individually (*n* = 3), and for the child at primary school there was a teacher present. Interviews/groups lasted between 30-120 min. Following the interview, participants were given the opportunity to share further information, thanked for their time and given a debrief sheet.

### Ethical considerations

Approval was given by the institution’s Ethics Committee (protocol number: aLMS/PGR/UH/03204(1)). Participant information sheets were adapted for each population, notably a child friendly version was made available for the young people. Prior to obtaining consent, participants were provided with this information and informed consent obtained from all participants; parents gave informed consent for their child to participate; and prior to the interview informed consent was also obtained from the children. Confidentiality was ensured throughout, and all participants were made aware of interviews being recorded, stored, transcribed by the first author, deleted on transcription. Identifying details were removed and participants consented to anonymised data being used for publication. Following the interview, participants and children’s parents were provided with contact details for additional support.

### Data analysis

A “quality reflexive” thematic analysis (Braun and Clarke 2019, p594) was conducted by the first author utilising an inductive process (Braun and Clarke 2006; 2019). All transcripts underwent initial line-by-line coding which were independently sorted and grouped to generate themes. To enhance validity and a more nuanced understanding of the data, separate coding was conducted by a peer, generating a reflexive conversation with the first author. As a flexible, iterative and inductive approach, uninfluenced by pre-existing ideas or frameworks, some themes were discarded, some merged, and others became subthemes or themes. Within this analytic process, the first and second authors repeatedly met to collaboratively reflect on the coding and the generation of themes within the three populations, developing a rich nuanced understanding of the data. This process highlighted convergences in the individual population theme tables. Whilst also reflecting on divergent narratives, the groups were merged to create one coherent thematic structure. Given the inclusive nature of the research strategy, and the lived experience within the team, reflexivity through reflective notes and supervision was used to acknowledge potential interviewer and researcher bias (O'Brien *et al.* 2014).

Credibility and inclusive research was further ensured through member checking on the final themes, corroborating these. The final assurance of inclusive research was the involvement in the research and paper authorship of a parent with lived experiences of educational support for a young person with ASD, who was also a clinician. To facilitate triangulation the research team also included a researcher experienced in neurodevelopmental conditions, a doctoral research student/clinician and clinicians working with neurodevelopmental conditions. In this process, authors agreed the final presentation of themes and the quotes used to represent those themes within this paper.

## Results

Analysis of the interviews resulted in the following main and sub-themes (Table 2).

**Table 2.** Main themes and subthemes.

Main themes	Subthemes
A system overwhelmed by unmet needs	A difficult system with inaccessible provision
	Funding issues and resources
	A constant battle with, and within, the system
The impact on quality of life	Not understanding others’ perspectives
	Lack of support negatively affects everyone
	Stagnation in life: feeling stuck
Hopes for the future	The value of support and external resources
	From alone to together
	Empowering people in the system

### A system overwhelmed by unmet needs

Fuelled by funding issues, parents and school staff highlighted a constant, exhausting, overwhelming battle to locate and access support.

#### A difficult system with inaccessible provision

Many parents believed coordination within schools was poorly set up, quickly breaking down and leaving them isolated and unsupported:

…Her class teacher didn’t even turn up to the handing over meeting…school were disengaged and unorganised…so I just felt like…here's a diagnosis, here's a giant boot, off you go. (Parent5)

Many parents felt bewildered and broken that there was no joined up thinking after diagnosis, often being left with no additional support:

…that broke me more than the day he got diagnosed because it was just like someone going, your child has this thing, we spent the whole summer trying to understand it…but he’s not going to get any extra support. (Parent4)

Likewise, staff were impacted by the unpredictability of ASD: *‘…you think “oh my Gosh, yes I’ve had a really good day”, the next day you come in and just like “oh gosh yeah, it’s really hard. So hard.”’(TA2).*

Further, staff reflected on the mismatch between expertise and provision, for example, SENCO4 emphatically stated: ‘…*people that…are expected to work with the…children with the most significant need, are the people who are the least qualified. Fact…and that’s not ok.’*

Even teachers did not understand how the system worked*: ‘Even the funding I have no idea how that works.’(Teacher1).* This extended to more senior staff with SENCO4 stating: ‘*I have not got a clue what money or what budget is going on. It’s all as far as I’m concerned completely fictional, means absolutely nothing.’* From the parents’ perspective, some thought the barriers to accessing provision may have been deliberate: ‘….*trying to access it for yourself or for someone in your family is a nightmare, and I am beginning to think that there are certain barriers that are deliberately put there.’(Parent6).*

#### Funding issues and resources

Alongside this inability to meet needs were concerns around financial restrictions hindering appropriate provision, potentially impacting on how resources were provided: *‘It is just resourcing and funding…it is as much support as our SENCO can give us…she can’t just have a money tree and give us everything we want, so it’s quite difficult.’(TA3).* Staff seemed aware of the flaws in the system, and the impact this had on appropriately meeting children’s needs:

…you…have a child now whose autism….he didn’t have the resources that he deserved a good couple of years ago and now he’s really struggling and behaviour is now coming out….he’s been excluded twice a week. He’ll end up in a PRU [*Pupil Referral Unit*] but genuinely hand on heart that’s not where he should be, that’s the autism not being resourced well enough and we just can’t manage it. (SENCO1)

Most parents were understanding of the school’s perspective:

I understand it's difficult for school as I know there's a lot of kids and I know that everywhere is stretched, its lack of resources, lack of time, all the time…they just can't do it, there isn't enough resources, there isn't enough teachers and schools are stretched too much to the point where it's just not working, so you know the budget gets cut every year. (Parent1)

#### A constant battle with, and within, the system

Given the pervasive lack of resources perhaps it was unsurprising that, in trying to meet their child’s needs, parents battled with the system:

It was very isolating in the beginning…you have to be proactive to fight for your child. You have to fight, is the word, to get basic needs…it is a fighting battle, but we all have to fight with it. (Parent3)

Parents described how exhausting this was, and the subsequent impact this had on them and their families:

It was the biggest struggle for me…that I just had no stamina left to say no and fight more…fighting for the right help puts a lot of stress on the family. (Parent8)

Pushing for resources whilst being pushed around seemed to become an aversive experience for parents:

I think you have to be really pushy, I mean you see some of the parents that come to the groups in tears because they’re getting pushed around and don’t know where to go. They’re getting really bullied by staff. (Parent2)

For staff, the battle also occurred with the system, demonstrating their concern when children got lost in the finances:

It becomes a little bit of a battle between you and the local authority…it’s not nice if…you’re sort of haggling over a child…and the child doesn’t become a child any more it becomes just….you need to give us more money, It becomes quite sort of you versus us. (SENCO2)

## The impact on quality of life

A lack of provision impacted all areas of life for parents, families and staff.

### Not understanding others’ perspectives

The ability to understand each other’s perspectives was frustrating and, for parents, compounded by punitive rather than understanding responses towards their children:

Teachers and TAs don't really take on board what it's like…I know they say they've worked with them…but there's certain things…for instance when they start giving out bad behaviour letters. (Parent1)

Communicating with people who did not understand made parents feel unheard and judged across organisations and systems:

I would say that the council and MPs are not helpful at all because I went to see my MP and they were just so judgmental…They were not listening to me and no-one is trying to understand how difficult it is for us. (Parent8)

For staff on the frontline, they felt communication focussed on demands: ‘*they [parents] can come in on us a bit harder and be like “put this in place, put that in place”’(Teacher2).*

In general, poor communication between schools, parents and wider systems left parents feeling invisible: ‘*They see parents as invisible people. We don't exist. No matter how many emails I send, no matter how many calls I do, I'm invisible’(Parent8).*

This extended to staff who were also negatively impacted by poor communication with the wider system: *‘I’m feeling undermined and let down to be honest…it is let down in communication’(Teacher3).* For parents, once their child was diagnosed, poor communication also occurred between services:

Social services and CAMHS [*Child and Adolescent Mental Health Services*]…It’s really weird, even though they’re working in the same building, they were not accessible to each other…Why are you not talking to each other? (Parent7)

In general, poor communication and misunderstandings fuelled negative relationships throughout the system, impacting everyone.

### Lack of support negatively affects everyone

Inadequate support negatively impacted mental health, physical health, and overall quality of life:

Because of the situation, my professional life suffered a lot, my personal life, social life, everything. It was not because of how my children were behaving…I was not having any support. (Parent8)

Many parents saw their child’s mental health severely impacted by the inability of systems to meet their child’s needs:

They were just at breaking point…you see your young child having a mental health crisis and it’s messed up…at five years old, how did it get to that point?…I had someone sort of go to me, well five-year olds can’t really have mental health problems, well…watch my child. (Parent4)

Many parents mentioned the significant impact it had on their own mental health:

Stress levels have gone through the roof…Evidence of that is my smoking's increased…I find myself quite annoyed, I feel that annoyance. I’ve become more aggressive towards people, short fuse, not taking it. (Parent7)

Most parents acknowledged they were unable to take proper care of their own physical health:

My physical health went where I couldn’t even walk…my physical health my mental health has to suffer because I have to make sure that I’m the primary carer for her because there’s no-one else to help. (Parent3)

This created tensions in their own health-care appointments: ‘*I had the GP sort of having a go at me, well you need to come in for this blood test…“Are you gonna watch my autistic son while I go and do this?”…health-wise it’s a huge thing’(Parent4).* Staff also discussed the profound impact a lack of support had on them:

I’ve had quite a few experiences where I’ve had children lash out at me, I’ve walked around with a black eye and it’s hard. Cos it affects your life as well, and sometimes you don’t feel like you’re doing enough because something isn’t working…There’s not enough of me to just go around and I’m exhausted by say lunchtime and I’ve got nothing to give, I’m empty. Empty vessel. (TA1)

Many staff felt personally impacted by restrictions in the system limiting their ability to support children:

…quite upsetting actually because I feel like I should be doing my job and I can’t do it. You feel like you’re letting the child down. So it…makes you beat yourself up a bit…it hits you heavy I think sometimes. (TA1)

In contrast, the children remained unaware of the wider struggles within the systems around them. One young person who had extensive early support had recently transitioned successfully to secondary school, and repeatedly reiterated:

Interviewer: Have you ever wished that you had more help?

CYP3: No, I'm fine with my help.

Perhaps in this context, the parents and staff’s efforts successfully supported some children, though perhaps at the expense of themselves. For example, SENCO2 stated: ‘*We are just sort of dogs really and… you know like we are bashed about in the press, everyone thinks they can have a shot at education cos everyone’s been through it’.* One staff was also a parent of a child with ASD and understood the impact provision had on attaining potential, or not:

I had a son with autism and he was doing really well, he come out with the highest grades in Year 6 SATS…As soon as he went to secondary school he is now not even going to achieve any grades at GCSE because they have just left him to sit quietly in the corner. And no matter how many times I go backwards and forwards….they don’t care. (TA4)

### Stagnation in life: Feeling stuck

Many parents reported a systemic lack of support meant they were unable to move on in life or in their career:

I was a senior civil servant…I used to deal with ministers, I had a proper job…I see people on LinkedIn and off they go with their careers…only now lots of years later has that sort of hit me about how…personally…that bit is just sort of erased. (Parent5)

This also impacted on parents’ ability to relocate geographically:

I would like to live out of London but it stops me from moving because I know that [local area] is the best place for them at this present time…obviously it's very nice to move out but you won't get the support that's needed…so it does stop people moving around which is a shame really. (Parent1)

Staff also felt stuck, often due to systemic misunderstandings about ASD in schools and the work required to meet needs:

With performance-rated pay…I’ve worked in a school where ninety percent of my children had to be at the expected standard at the end of Reception…honestly it’s not gonna happen, so you don’t go up the pay scale the next year, and you’re just stuck in this like really unpleasant catch twenty-two. (SENCO2)

### Hopes for the future

Although people’s experiences were overwhelmingly negative, some positive examples emerged, often through external provision.

#### The value of support and external resources

All parents had positive experiences of receiving support from the local parent charity:

This place has been a godsend…what [they] provided me with is more than what anyone else has provided me with…Meeting other parents, information, knowledge, open…a place where you can come if you've got problem, that is client-centred…they gave me an environment where they can listen to me. (Parent7)

Some schools were able to focus on the needs of each individual child, which seemed easier to attain in primary schools, particularly when children and provision were provided early in the child’s schooling:

…if they come in earlier…on in the school, that’s when we make a big difference but when they come in half way through it’s difficult to kind of fit them in. But when they come in young you see the biggest change. (Assistant Head)

There were also some examples where parents had positive experiences of school support:

School is brilliant, they've got a home-school communication book, which can go in both directions…we get reports back in the book about what he did that day…it's all really good…any time I've raised an issue they've dealt with it really quickly. (Parent6)

Children also talked about positive experiences of support they had received in school:

CYP1: When I'm stuck on my work…They explain it to me.

Interviewer: Who explains it to you?

CYP1: My teacher, and then my teacher assistant…they help me figure out what I'm meant to do.

For staff, there was one overwhelmingly positive source of support – the local specialist school outreach service who: ‘*come in and they give us support as and when we need it…So they help us move forward which is really helpful’(TA4).* Such support seemed vital:

I think they are absolutely amazing…drop of a hat they’ll come and help and they’ve supported us massively with some really high need pupils…they’ll…be like “you are doing the right thing” and you feel great…Giving you practical resources…they are just an absolutely incredible resource and I think that every local authority should have one. (SENCO2)

#### From alone to together

Many parents described a lone journey where they had to take sole charge: ‘*I'm more proactive myself so everything that I've done I've done myself’(Parent1).* For some parents, this even meant setting up their own support groups which helped bridge a gap and connect with others:

I made a support group. So, we all are together, we all like put our…problems there, and…listen to it, and help each other, advise each other, no professionals. So, this is the best thing so far because I learned a lot from them. I am still learning. (Parent8)

Given the lack of support elsewhere, most parents felt they had to support each other:

I think parents have to pull together in a big way to make a difference because you just can't leave it now to outside agencies…to do things, you have to…make your own support group, talk about things together. (Parent1)

#### Empowering the people in the system

For the children interviewed, ASD adjustments had happened earlier on, and consequently, their awareness of ASD related issues at school was minimal: ‘*Actually I've never had really any big problems’(CYP3).*

For parents and staff navigating the system, many recommended systemic changes to solve the issues they had experienced. For example, most parents believed schools should have an important role in promoting awareness around ASD:

Sometimes other parents are so upset….they think I'm not stopping my child. So, if there is enough awareness in the school and they do enough assemblies or send…information in the newsletters, parents should be aware…that some children are different. They don't do it on purpose. (Parent8)

Staff agreed more was needed and acknowledged that the wider community could sometimes have a large negative impact:

…the children suffer, and the parents suffer in the wider community because when they are in the shops and their child is doing things that other adults don’t understand, they are either insulting the parents or being rude to the child so actually there’s the bigger and wider community that makes the impact. (Assistant Head)

Many staff advocated whole school approaches to ASD, believing this would help all pupils:

Autism peer awareness - just do it with every class, just try and make it…always a school approach…make that a school policy, every class does it cos it’s going to work for everyone…you just run that as whole staff training because it’s not just children with autism that are going to benefit from it - make that an absolute standard in your school. (SENCO2)

Most staff believed all teachers needed to receive more appropriate training to support pupils with ASD:

I do wonder if we’ve…lost something having the year out training or two-year course. My course was very intense SEN. I learnt so much in that year and I just wonder if…we’re perhaps not preparing our staff enough. We can’t learn it from one lecture. (SENCO3)

Staff also identified ways the system could change to better meet the needs of children with ASD:

Like the resources you could use in class. They’re not readily available for every single person. I think it would be good if everyone had a set of whatever it is you needed…if it was…more widely spread. It is just resourcing and funding. (Teacher3)

One participant with ASD was able to articulate differences in the quality of support given by different staff and through this identified the key factors in TA work which supported them, or not:

Interviewer: How does she treat you like a child?

CYP4: Like always comes towards me…I do the work, she always comes and starts annoying us, mostly me, and that really ticks me off, and I'm…going to be honest with you I do act very aggressive and tell her to go away, so I get really aggressive towards her and I really don't like her

Interviewer: What could she be doing differently that would make it better?

CYP4: Um I dunno maybe don't come near me and don't get me annoyed…she's just only in science. The other two TAs are perfect, they're real good…I feel like I can trust them

Further, those who had successfully transitioned to secondary seemed settled with friends and had developed an ability to identify solutions or assert themselves. For example, when discussing bullying:

CYP3: Secondary school (bullying) went up more but not that much…apparently one person called me…actually I've no idea what they called me, but I definitely got bullied. Happened yesterday, but you know what, I was like, “I really don't give a shit!”…They try to bring me down but, no, that's not going to happen, ever.

Most children stated breaks were important, with one recommending: ‘*If the class gets too loud I'd like to have like a minute or so outside…Well just you know make the break and lunchtimes a little bit longer, that's all’(CYP2*). Noise and fatigue from work necessitated longer breaks and access to a quiet room to rest: ‘…*this room is usually open as a sort of moment of quiet…I'm usually able to rest my mind*’*(CYP2).*

Many parents discussed how they would like to see children with ASD receiving regular follow ups via an ongoing service*:*

I’d like….the people that are involved in the diagnosis to follow your child so that you see them six monthly or even yearly intervals and they come into school and they do this thing of saying how’s it going, what could be better, here’s our top tips from people who understand autism and know a bit about your child. (Parent5)

Crucially, with support in place, the children had developed a resourcefulness and voice, seeing potential and resilience in their future:

…my advice would be….if you got autism take it as like a superpower….for example, Black Panther or Iron Man or Super Mario or other video games characters, take it like you have this power to change the world or…you can use it for creativity, or do whatever you want with it. Even if life brings you down, you can always put it back up…and maybe, if you're lucky, you can be successful with your autism. (CYP4)

## Discussion

Once diagnosed, layers of relationships with individuals, groups, or organisations (Bronfenbrenner 1992) protected the children in this study from stresses within the education system, making them feel supported and included. Having successfully integrated into school, they believed they, and their parents, received enough support.

In contrast, parents’ journey navigating the system to access this support was overwhelming. As the child’s main source of support, parents talked about the negative impact minimal support had on their child (Murphy *et al.* 2016), their own mental and physical health, and their ability to progress in their lives (Dillenburger *et al.* 2015; Vasilopoulou and Nisbet 2016). Managing this was stressful and perhaps explains why parents of children with ASD experience high levels of stress (Dillenburger *et al.* 2015; Vasilopoulou and Nisbet 2016).

Another key relationship for the child were teachers tasked with directly supporting the child and coordinating provision. Many parents were frustrated by poor communication from schools and professionals, presenting a considerable barrier to accessing support. Equally, staff felt parents’ high expectations put excess pressure on them; the battle which ensued often negatively impacted parents’ relationship with wider systems (Hebron and Bond 2017; Galpin *et al.* 2018; Preece 2014). For most parents, this dynamic was their main battle ground reflecting a pervasive fight to access support (Crane *et al.* 2018; Galpin *et al.* 2018).

Extending the literature, staff also battled the wider system describing how additional pressures impacted their mental health and career progression, presenting a quandary for those dedicated to facilitating the inclusion of children with ASD. Here, the findings from both parents and staff were that schools struggled to meet the needs of some pupils with ASD, with funding and resource issues being a key barrier (Lindsay *et al.* 2013; The Key 2016b; National Autistic Society 2016).

In this void, other parents became the main sources of informal or formal support for parents through voluntary sector organisations; and for staff in this region there was a specialist outreach service which successfully filled gaps. Interestingly, a decade ago schools were a positive source of support with parents generally satisfied (Whitaker 2007; Parsons, Lewis, and Ellins 2009). Aside from discussions around the outreach service and parent support groups, this was not replicated in parent or staff dialogues, highlighting the likely impact from a decade of budget cuts, potentially explaining the emotional toll this was taking.

Operating in the background was the larger system - the local authority and local community - which impacted those surrounding the child (Bronfenbrenner 1992). With inadequate knowledge and understanding about ASD (Galpin *et al.* 2018; Preece 2014), parents were disappointed (Roberts and Simpson 2016), believing staff had limited knowledge (NAS, 2017) which was also recognised by staff (NASUWT 2013). Indeed, staff identified their own lack of expertise, with funding and training issues impacting access to ASD-specific training making them inadequately prepared (Symes and Humphrey 2012; Lindsay *et al.* 2013). Many of the parent and child participants had experienced bullying or discrimination. Further, staff and parents reported examples of children who had slipped through the net and were struggling. Perhaps this stemmed from societal values, attitudes and laws influencing dominant narratives around neurotypical development, but also schools favouring high attaining students (Hedegaard-Soerensen and Grumloese 2020).

The experiences of accessing support negatively impacted on parent’s wellbeing (Camm-Crosbie *et al.* 2019), and teacher wellbeing; yet by protecting the children who had received earlier continued support and were managing in school, these children seemed buffered from its impact. Consequently, the children interviewed learned to defend themselves amongst their peers, with many reporting positive experiences and friendship groups. Indeed, the children embraced their ASD and seemed content by the dialogues and education around this.

A strength of this study was the triangulation of findings through interviews with parents, staff and children; and consultation from parents, teachers and children occurring throughout the project. Nevertheless, the teachers who participated may represent a small section of staff dedicated to supporting parents and children with ASD to access mainstream education and may not be representative of other mainstream teachers. Further, parents were recruited from one local parent forum; thus, parents who were more isolated or marginalised are not represented. Another limitation was the small sample of children who had earlier provision and felt supported. Children who were struggling in school, or not attending school were not represented, and it is noted that some parents and staff highlighted this alternative narrative. Additional research is needed with children with ASD, to help identify areas of difficulty, particularly those who have slipped through the net, and expand on ideas around how to help meet their needs moving forward into adulthood. Finally, given the reflexive qualitative nature of the analysis, to enhance inclusivity practices and measure outcomes of adjustments for young people with ASD, quantitative research is required to provide greater specificity around key priority areas. This will help guide the development of recommendations for practice within school settings. Qualitative research utilising alternative reliability methods in the coding process could be used to further elucidate understanding in this emerging evidence base (O’Connor and Joffe 2020).

## Conclusion and recommendations

It seemed children were protected and clearly felt a sense of belonging at school (Goodall 2020), however, parents and staff reported experiences of systemic discrimination. Buffering against inequalities within organisations and policies was exhausting, yet seemed the only way to obtain necessary provision; perhaps representing structural inequalities in the system (Magana *et al.* 2012). To alter negative trajectories for children with ASD, support should be provided early and proactively from education and healthcare, and should be maintained even when children are managing, rather than provided reactively when children are struggling or withdrawn when they are managing. Clearly a systemic issue requires multi-systemic solutions (Bronfenbrenner 1992) and challenging the status quo by developing existing strengths, like the concept of ASD as a superpower, would enable those on the frontline to maintain their wellbeing for the longer-term benefit of the children. Inclusive research provides opportunities to focus on strengths (Walmsley, Strnadová, and Johnson 2018), and staff seemed committed to this practice, but collaboration with parents is essential (Roberts and Simpson 2016; Hebron and Bond 2017) and resources are required to implement multiple strategies (Lindsay *et al.* 2014; Hebron and Bond 2017).

To encourage inclusivity, implementing a centralised outreach service for schools and parents in all regions should be a prioritised; pooling resources into one place would facilitate earlier recognition and adaptations for ASD through signposting, the dissemination of ASD awareness in local communities and training for all teachers. This would also facilitate the implementation of a school-wide Autism Competency approach (Roberts and Webster 2022) which would benefit all pupils.

Furthermore, due to the focus of higher management on examination results, SEN pupils have the lowest priority for teachers. Given teachers with the greatest skills work more effectively with the pupils with the highest needs (Humphrey and Symes 2013), TAs should work with those who need less support and specialist teachers work with ASD. This would require a conceptual shift and support from the wider system of headteachers, SENCOs and Ofsted. To support inclusion for all children with ASD, it is critical this begins to change (Lindsay *et al.* 2013). Thus, policy makers, commissioners, and all staff who work with children with ASD should prioritise systemic changes, on several levels, to improve this situation.
